# Congenital vaginal atresia: about an uncommon case

**DOI:** 10.11604/pamj.2020.37.69.21682

**Published:** 2020-09-17

**Authors:** Aziz Slaoui, Intissar Benzina, Sarah Talib, Amina Etber, Najia Zeraidi, Amina Lakhdar, Aicha Kharbach, Aziz Baydada

**Affiliations:** 1 Gynaecology-Obstetrics and Endoscopy Department, Maternity Souissi, University Hospital Center IBN SINA, University Mohammed V, Rabat, Morocco,; 2 Gynaecology-Obstetrics and Endocrinology Department, Maternity Souissi, University Hospital Center IBN SINA, University Mohammed V, Rabat, Morocco

**Keywords:** Congenital vaginal atresia, hematometrocolpos, vaginoplasty

## Abstract

Congenital vaginal atresia is a rare congenital abnormality of the female reproductive tract due to a failure of canalisation in the urogenital sinus. We report the uncommon case of a 14-year-old girl with a primary amenorrhea associated to a cyclical pelvic pain, in whom examination objectified a vaginal cup that replaced the introitus. Ultrasound examination and magnetic resonance imaging (MRI) revealed atresia of the lower third of the vagina. The diagnosis of partial vaginal aplasia on functional uterus was retained, the patient had a perineal vaginoplasty. The evolution was satisfactory with regular cycles and improvement of pelvic pain. The decline is three years. Congenital vaginal atresia is a rare malformation classically and clinically pictured as a primary amenorrhea with chronic cyclic pelvic pain. Diagnosis is based on clinical examination and imaging. The MRI is designed to assess the importance of atresia and guide surgical management while the surgical technique aims to restore the integrity of the utero-vaginal tract and to increase the possibility of pregnancy for these patients.

## Introduction

Congenital vaginal atresia is a rare congenital abnormality of the female reproductive tract due to a failure of canalisation in the urogenital sinus [1]. During embryogenesis, the Müller's ducts form the tubes, the uterus and the upper two-thirds of the vagina while the lower part of the vagina depends on the urogenital sinus. Isolated vaginal aplasia is therefore the consequence of a defect in the development of the terminal part of the paramesonephrotic channels [1]. Its incidence is 1/4500 female births [2]. Most medical category systems do not consider it or include it in their classification especially because of its rarity. As an example, the American Society for Fertility Medicine considers complete vaginal atresia as a part of Mullerian agenesis and dysgenesis [2]. We report the case of an isolated vaginal aplasia on functional uterus and discuss the clinical and therapeutic aspects of this uncommon malformation.

## Patient and observation

**Figure 1:**
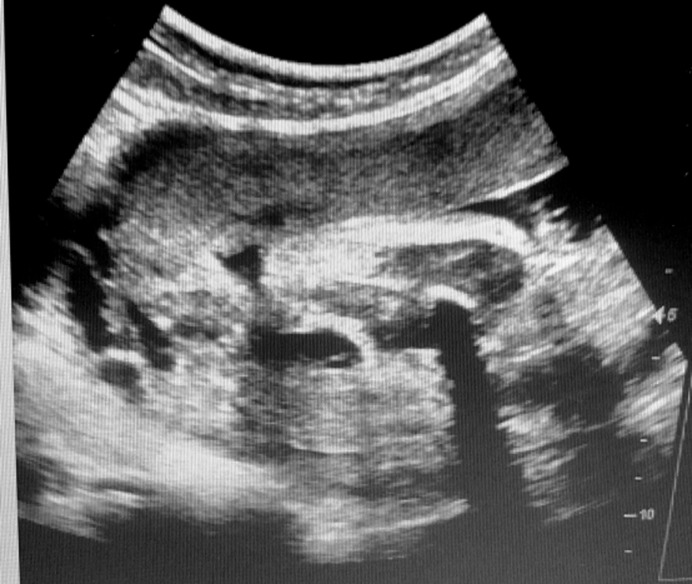
ultrasound appearance of the hematometrocolpos extending to the upper third of the vagina

**Figure 2:**
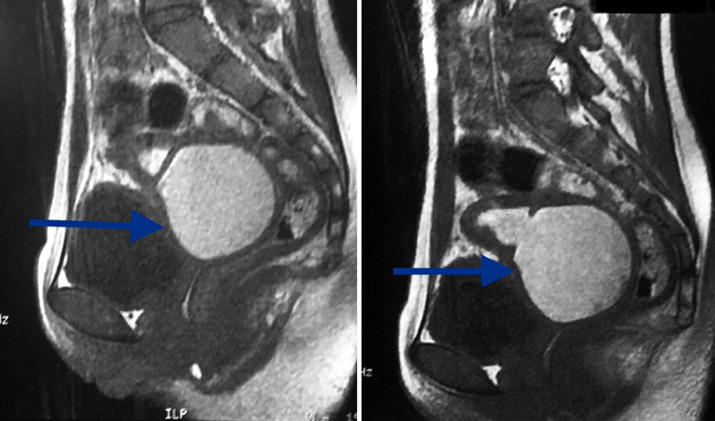
MRI appearance in T2 sequence of vaginal atresia with hematometrocolpos

We hereby report the uncommon case of a 14-year-old girl patient with no history of any particular disease who was seen for primary amenorrhea associated with chronic and cyclic pelvic pain that had been evolving for six months. Clinical examination revealed a normal morphological type, good size and well-developed secondary sexual characteristics, with the presence of a hard and painful pelvic mass of 3 cm width and 2 cm height. At the vulvo-perineal inspection, there was a normal appearance of the external genitalia with perfectly defined labia majora, labia minora and clitoris. At the vaginal examination, there was a vaginal cup instead of the introitus, with the perception of a painful pelvic mass across in favor of hematometrocolpos. Ultrasonography showed a normal-sized uterus with the presence of normal ovaries, tubes, cervix and a fluid retention at the emptiness line of the uterus continuing into the upper third segment of the vagina in relation with a hematometrocolpos and an absence of the lower third segment of the vagina (Figure 1). Pelvic MRI showed an anteverted uterus of 91 mm in height, 49 mm in thickness and 55 mm in width, with fluid retention at the emptiness line of the uterus continuing into the vaginal cavity. It measured 43 mm height, 21 mm wide and 17 mm deep and was probably in favor of a hematometrocolpos. The junctional zone was thickened to 12 mm and the myometrium was homogeneous, with presence of normal tubes and ovaries. The cervix was dilated by the retained fluid but had normal morphology and 44 mm of length. The lower third segment of the vagina was not seen on the MRI (Figure 2). Hormonal analyzes were within normal limits and her karyotype showed 46XX. The diagnosis of congenital vaginal agenesis on functional uterus was retained.

**Figure 3:**
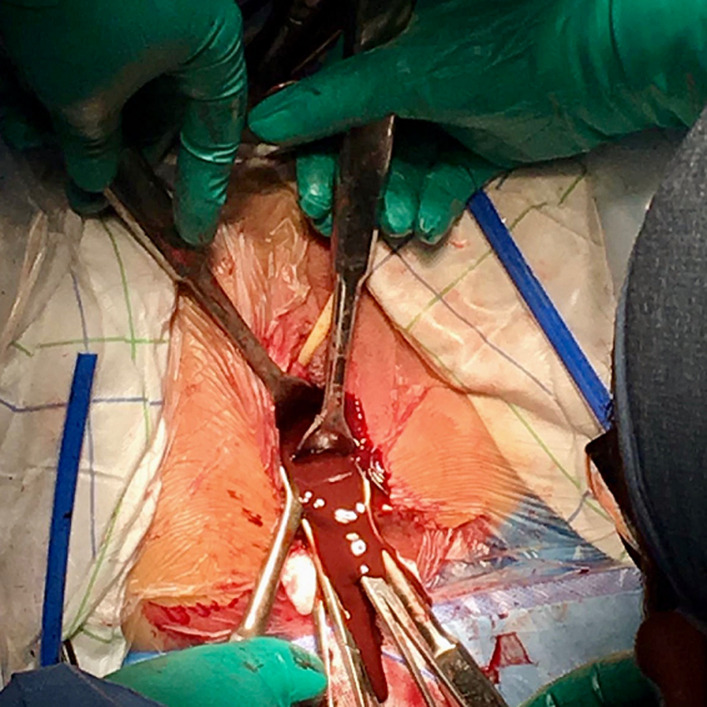
per-operative image of the drained hematometrocolpos

**Figure 4:**
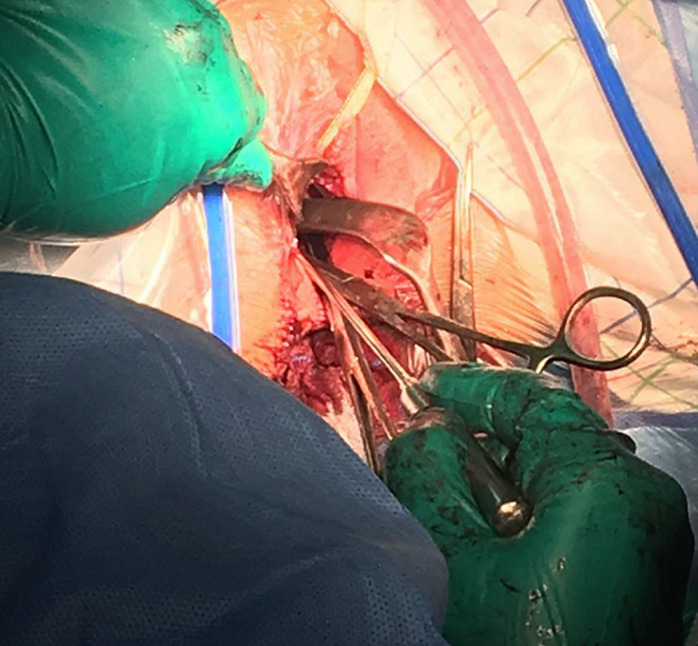
per-operative image of the hysterometer through the neovagina

Prior to the operation, she received an informed consultation on the prognosis of her fertility. A verbal and written consent for the procedure was collected from her legal guardian. The patient underwent then a perineal vaginoplasty. The technique used consisted of vaginal traction of the upper two-thirds of the normally formed vagina followed by subcutaneous fixation, thus forming a neo-introitus. We began with a transverse incision of the vaginal cup. We dissected the fibrous masking material until the normal vagina was reached. At this point the collected blood was drained (Figure 3). The introduction of the hysterometer allowed us to check the uterine emptiness and the normal length of its bottom (Figure 4). The normal vaginal mucosa was then drawn and attached to the subcutaneous border by single points spaced by 5 mm each in a radial disposition. The new introitus obtained was kept open by silicone vaginal probes lubricated with estrogen gel until re-epithelialization occurred. Blood loss was quantified at 150 ml. The operating time was 114 minutes. The cicatrisation was completed in 4 weeks. The evolution was marked by the appearance of regular cycles with marked improvement of pelvic pain. The nine-year retreat allowed us to review a 23-year-old married woman with a newborn baby boy and a fully satisfied sex life.

**Consent for publication:** written informed consent was obtained from the patient for publication of this case report and any accompanying images. A copy of the written consent is available for review by the Editor-in-Chief of this journal.

**Ethics approval and consent to participate:** ethics approval has been obtained to proceed with the current study. Written informed consent was obtained from the patient for participation in this publication.

## Discussion

Total or partial isolated vaginal atresia is a rare congenital anomaly. It represents only 9% of cases of vaginal aplasia [3]. Most of it is usually associated with Rokitansky´s syndrome. The cervix may be normal or atresic. This malformation is secondary to a defect in the development of paramesonephrotic ducts during embryogenesis. The vagina is then replaced by a fibrous tissue [4]. The common clinical picture is that of a girl in a pubertal age who consults for primary amenorrhea and whose normohormonal character is evident from the inspection that shows normally developed secondary sexual characteristics as in our patient. The interrogation raises the notion of cyclic chronic pelvic pain. The clinical examination finds a normal morphotype, a correct traille, a good mammary development, an axillary and pubic hairiness provided and perfectly constituted external genitals. The speculum examination associated with the vaginal touch or the monodigital vaginal touch combined with the digital rectal examination perceives only a vaginal cupule which measures 3 to 4 cm, this vaginal cup is one-eyed devoid of slits. The uterus is usually increased in size because of the hematometrocolpos. Rarely, the diagnosis may be late for a woman with primary amenorrhea but who consults for difficult sexual intercourse because of insufficient vaginal penetration or who comes for primary infertility consultation when the vaginal cup has been sufficiently distended by successive coitts to the point of allowing a normal sexual life [5]. Exceptionally, the diagnosis can be made in the presence of chronic pelvic pain in the neonatal period or prepubertal period with increased uterine volume in relation to a hydrometrocolpos secondary to the accumulation of mucus or endometrial and cervical secretions due to stimulation estrogen in the prepubertal period. The mucus thus accumulated can become infected and make a pyometrocolpos [5]. Dural *et al*. [6] reported the case of isolated vaginal atresia discovered as part of the assessment in a recurrent urinary tract infection of a prepubertal girl. Careful clinical examination can be used to make the diagnosis suspect and to avoid any unnecessary hormonal assessment or excessive hormone therapy.

Pelvic ultrasound confirms the diagnosis. It objectifies the image of a hematometry with total or partial vaginal agenesis, functional ovaries containing follicles. Renal ultrasound or intravenous urography will look for an associated urinary malformation. Moreover, the early recognition of a single pelvic kidney can avoid a possible trauma during surgical reconstruction. The advantage of MRI is to better specify the height and extent of vaginal aplasia as long as guiding the choice of the most appropriate surgical technique [7]. Vaginal atresia may be associated with malformative uropathies such as unilateral renal agenesis, ectopia, malrotation or renal dystrophy. It may also be associated with skeletal abnormalities such as scoliosis, vertebral fusion, syndactyl and thenar eminence hypoplasia. As it may be part of a polymalformative syndrome in the Winter syndrome that associates renal, genital and auditory malformations, or McKusick Kaufman's syndrome that associates postaxial polydactyly, cardiac malformation and hydrometrocolpos [7]. Our patient had an isolated vaginal atresia. No associated malformation was found.

The management of these patients is surgical [4, 5]. It aims not only to create a neovagin that would allow satisfactory sexual intercourse but also to restore a normal utero-vaginal pathway in order of allowing the evacuation of menstrual blood and cervical secretions. The establishment of therapeutic amenorrhea must be the rule in order to perform a precise morphological assessment and to prepare the girl for surgery [5]. The age of care is controversial. We believe that, in the event of a diagnosis established by chance during an imaging examination carried out for another reason in asymptomatic prepubertal patients, the vaginal reconstruction should be postponed to pubertal age as soon as the hematocolpos appears before installation of the hematometrocolpos. This approach will allow sufficient development of the vaginal tissue facilitating vaginal anastomosis in limited partial forms, in addition, it would reduce the risk of stenosis which is a frequent complication. There is no standard surgical technique, all the techniques have in common the realization of a preliminary dissection of the fibrous plane located between the bladder and the urethra forward and the rectum behind, and this, perineal or mixed perineal-abdominal, with however the risk of fake roads that is not negligible. One can thus help a digital rectal examination or candles introduced into the rectum [8]. The management of limited vaginal aplasia when the atretic segment does not exceed 3 cm is simple, it is done by direct anastomosis of the vaginal mucosa. In contrast, surgery of extended forms is more complex, it uses cutaneous or intestinal grafts [8]. Michala *et al*. [4] described three types of vaginoplasties. The first technique is so-called Williams vulvovaginoplasty; this simple procedure involves the labia majora in a perineal pouch. Although it is a relatively minor procedure and no longer practiced since it does not provide a satisfactory sexual intercourse with penetration. The second technique is the Vecchietti procedure, which consists of a gradual vaginal pressure that increases the size of the vaginal vault. The last group includes all the procedures that aim to create a neovagin within the rectovesical space and lined it with different types of tissue such as skin (McIndoe Reed), peritoneum (Davydov), intestine or - perhaps in the future - tissue engineered vaginal mucosa [9].

The goal remains the same: to produce a functional and aesthetically acceptable vagina, especially since unlike Rokitansky's syndrome, vaginoplasty in isolated vaginal aplasia can allow pregnancy [7]. Each surgical technique has its advantages and disadvantages, but many of them have now been replaced by laparoscopic procedures. This makes them less invasive, with shorter recovery times and avoids large abdominal incisions, potentially avoiding many complications [4, 9]. It is also a field of surgical expertise where robotic surgery takes all its interest [10].

## Conclusion

Congenital vaginal atresia is a rare malformation classically and clinically pictured as that primary amenorrhea with chronic cyclic pelvic pain. Diagnosis is based on clinical examination and imaging. MRI is designed to assess the importance of atresia and thus guide surgical management. Whatever the surgical technique, it will aim to restore the integrity of the utero-vaginal tract and allow these patients, whose psychological experience is particularly difficult, to have a satisfactory sex life and increase their possibility of pregnancy.
